# Increasing growth and yield by altering carbon metabolism in a transgenic leaf oil crop

**DOI:** 10.1111/pbi.13363

**Published:** 2020-03-18

**Authors:** Madeline C. Mitchell, Jenifer Pritchard, Shoko Okada, Jing Zhang, Ingrid Venables, Thomas Vanhercke, Jean‐Philippe Ral

**Affiliations:** ^1^ RMIT University Melbourne Vic Australia; ^2^ Food Agility Cooperative Research Centre Sydney NSW Australia; ^3^ Commonwealth Scientific and Industrial Research Organisation Canberra ACT Australia

**Keywords:** triacylglycerol, lipid, carbon partitioning, carbon fixation, CBB cycle, SBPase, FBPase, DOF4

## Abstract

Engineering high biomass plants that produce oil (triacylglycerol or TAG) in vegetative rather than seed‐related tissues could help meet our growing demand for plant oil. Several studies have already demonstrated the potential of this approach by creating transgenic crop and model plants that accumulate TAG in their leaves and stems. However, TAG synthesis may compete with other important carbon and energy reserves, including carbohydrate production, and thereby limit plant growth. The aims of this study were thus: first, to investigate the effect of TAG accumulation on growth and development of previously generated high leaf oil tobacco plants; and second, to increase plant growth and/or oil yields by further altering carbon fixation and partitioning. This study showed that TAG accumulation varied with leaf and plant developmental stage, affected leaf carbon and nitrogen partitioning and reduced the relative growth rate and final biomass of high leaf oil plants. To overcome these growth limitations, four genes related to carbon fixation (encoding CBB cycle enzymes SBPase and chloroplast‐targeted FBPase) or carbon partitioning (encoding sucrose biosynthetic enzyme cytosolic FBPase and lipid‐related transcription factor DOF4) were overexpressed in high leaf oil plants. In glasshouse conditions, all four constructs increased early growth without affecting TAG accumulation while chloroplast‐targeted FBPase and DOF4 also increased final biomass and oil yields. These results highlight the reliance of plant growth on carbon partitioning, in addition to carbon supply, and will guide future attempts to improve biomass and TAG accumulation in transgenic leaf oil crops.

## Introduction

Plant oil is important for human and animal nutrition as well as being a source of biofuels and chemical feedstocks for industry (FAO, 2012). Currently, most plant oil is derived from seed‐related tissues but there is increasing interest in genetic engineering of vegetative tissues to produce oil due to the potential yields from high biomass crops such as tobacco, sorghum and *Miscanthus* (Weselake, 2016). Several studies have demonstrated the feasibility of using transgenic plants to produce oil in leaves, for example achieving several fold‐increases in the accumulation of oil (triacylglycerol/TAG) in *Arabidopsis thaliana*, sugarcane, sorghum and tobacco through the overexpression of lipid‐related transcription factors and biosynthetic genes (Andrianov *et al.*, 2010; Sanjaya *et al.*, 2011; Vanhercke *et al.*, 2018; Vanhercke *et al.*, 2017; Vanhercke *et al.*, 2014; Zale *et al.*, 2016). Further increases in TAG content can also be achieved through silencing of competing pathways such as starch synthesis or TAG degradation (Sanjaya *et al.*, 2011; Vanhercke *et al.*, 2017).

However, TAG synthesis at the expense of other carbon‐rich compounds, particularly important energy reserves such as starch and sugars, has the potential to limit plant growth. While leaves have significant capacity for TAG synthesis, high turnover leads to low steady‐state levels (Tjellström *et al.*, 2015), so TAG is usually only a minor component of carbon stores (Chapman *et al.*, 2013). In contrast, most of the carbon fixed through photosynthesis is partitioned into soluble sugars and starch, which are accumulated and depleted during dark/light cycles. During the day, triose‐phosphates produced via the Calvin–Benson–Bassham (CBB) cycle feed directly into the synthesis of sucrose, which can be transported to other parts of the plant, while leaves can also store carbon in the form of transitory starch (carbon sink). These leaf starch reserves can then be degraded at night to fuel plant metabolism (carbon source). This balance between starch as ‘source’ and starch as ‘sink’ is key to plant development (MacNeill *et al.*, 2017) so competition for substrates between the starch/carbohydrate and TAG biosynthetic pathways could have unintended and far‐reaching effects.

In the first version of transgenic high leaf oil tobacco (HO; overexpressing WRINKLED1/WRI1, diacylglycerol acyltransferase1/DGAT1 and oleosin), TAG accumulates to around 15% of leaf dry weight, compared with <0.2% in the wild‐type (WT; Vanhercke *et al.*, 2014). TAG accumulation is also associated with a 75% reduction in the starch content of mature leaves, and final height is reduced in T2 HO plants (Vanhercke *et al.*, 2017; Vanhercke *et al.*, 2014). In second version tobacco plants, overexpressing the lipid‐related transcription factor LEAFY COTYLEDON2 (LEC2) as well as WRI1, DGAT1 and oleosin, a further doubling in leaf TAG content is associated with lower final biomass than HO plants and leaf starch content is strongly and negatively correlated with leaf TAG content (Vanhercke *et al.*, 2017). This suggests that there may be a trade‐off between TAG and starch accumulation and that high TAG accumulation may impair plant growth. However, these measurements were performed at only a single time point late in plant development and the differences were not fully explored.

The first aim of this study was thus to compare WT and transgenic HO tobacco plants and identify changes in carbon partitioning, metabolism and growth associated with leaf oil production. Given the apparent reduction in biomass in HO plants, the second aim was to overexpress additional genes related to carbon fixation and partitioning in the HO genetic background in order to improve growth and/or oil yield.

The first part of this study identified strong differences in HO plant growth, development and carbon partitioning compared with WT plants. HO plants were smaller at vegetative and flowering stages with a reduction in leaf sugars and amino acids during vegetative growth stages. It was hypothesized that low leaf sugar concentration may signal a low energy/carbon status to the plant and slow vegetative growth, so HO plants were supertransformed with one of four genes identified as potentially able to provide extra energy reserves for TAG synthesis and/or plant growth. Specifically, these genes were intended to increase carbon supply (encoding CBB enzymes sedoheptulose‐1,7‐bisphosphatase/SBPase or fructose‐1,6‐bisphosphatase/FBPase), leaf sugars (cytosolic FBPase) or other important carbon‐rich metabolite pools (lipid‐related transcription factor DOF4).

Briefly, the rationale was that overexpression of SBPase and chloroplastic FBPase has already been shown to increase carbon fixation and boost leaf hexose, sucrose and starch content (Lefebvre *et al.*, 2005; Tamoi *et al.*, 2006), which in turn could support high TAG synthesis while maintaining enough starch and carbohydrate reserves for growth. Alternatively, overexpression of cytosolic FBPase, which plays a key role in sucrose biosynthesis (Serrato *et al.*, 2009), might improve growth by increasing available energy reserves and/or influencing sugar signalling. Finally, increasing the efficiency of TAG synthesis by overexpressing the lipid‐related transcription factor DOF4, which increases oil accumulation in *A. thaliana* and *Chlorella ellipsoidea* without impairing growth (Wang *et al.*, 2007; Zhang *et al.*, 2014), might recover some lost carbon for carbohydrate metabolism and/or growth.

In glasshouse conditions, overexpression of each of these four genes in the HO background increased vegetative growth while overexpression of chloroplast‐targeted FBPase and DOF4 also increased final biomass and oil yield. These results highlight the importance of carbon partitioning to the successful development of metabolically engineered oil crops and will guide future attempts to create and refine higher biomass, higher yielding plants.

## Results

### Reduced relative growth rate and biomass in high leaf oil tobacco

**Figure 1:**
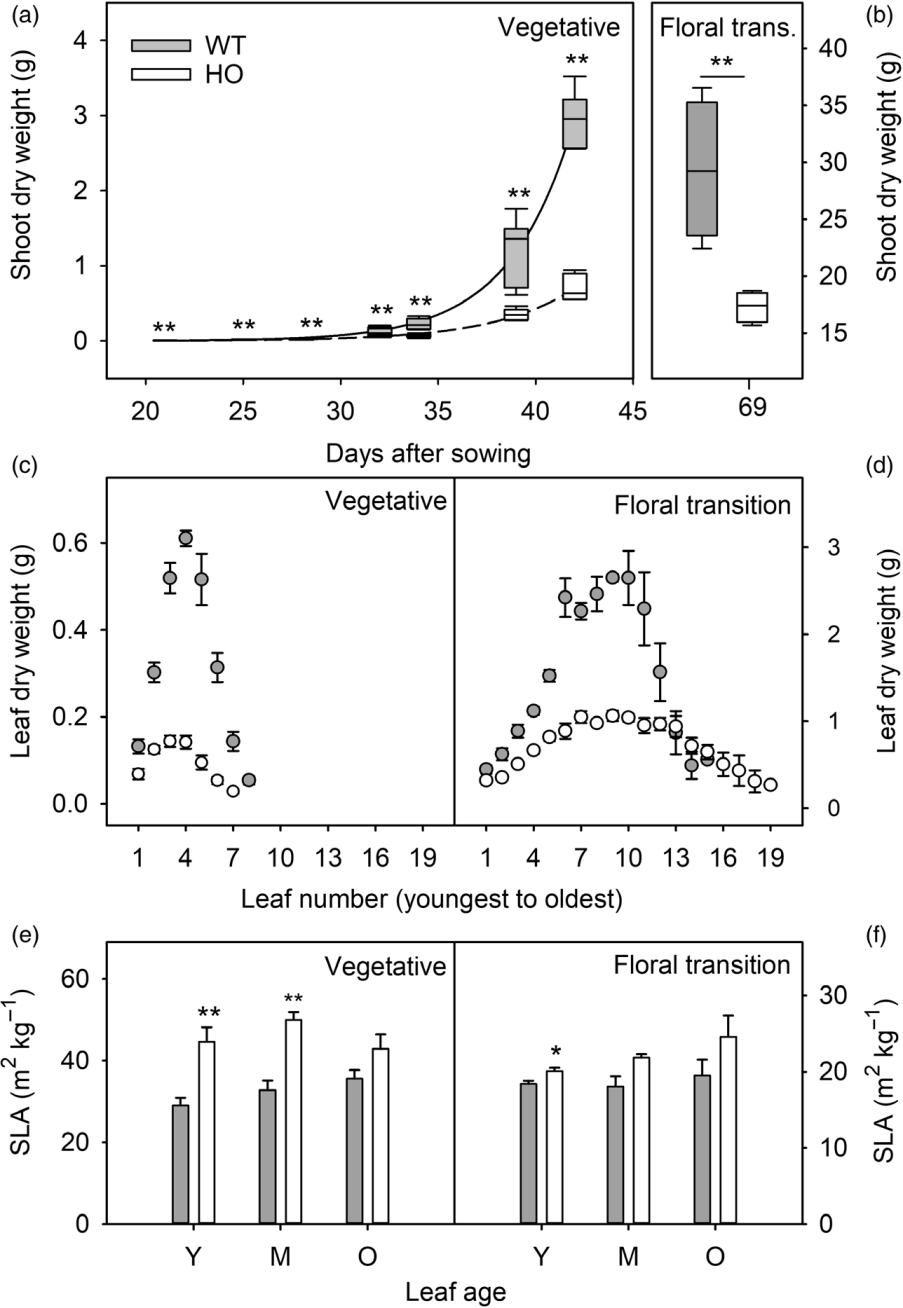
Growth and biomass allocation of WT (grey) and transgenic HO (white) tobacco plants. Shoot dry weight (leaf and stem mass) is shown for plants harvested from 21 to 42 DAS (vegetative stage: a) and 69 DAS (floral transition stage: b). Individual leaf dry weights (c, d) and specific leaf area (e, f) are shown for plants harvested at either the vegetative (c, e) or floral transition (d, f) stages of growth. Specific leaf area is shown for young/expanding (Y), mature/fully expanded (M) and older (O) leaves. Errors bars represent the standard error of 4–6 plants. Asterisks represent statistically significant differences (*P < 0.05; **P < 0.01) between the two lines according to a *t* test.

To quantify differences in growth and biomass allocation in WT and HO tobacco plants, the relative growth rates of WT and HO plants were determined by measuring aboveground dry biomass at different time points during the vegetative stages of growth (Figure 1a). WT plants were significantly larger than HO plants (*t* test, *P* < 0.01) at all stages of growth from 21 to 42 DAS. At 42 DAS, HO plants were approximately 25% of WT size, mostly due to smaller and fewer leaves. WT plants had a mean of eight large leaves whereas HO plants had only seven smaller leaves (Figure 1c). While WT plants maintained a growth advantage into the floral transition (69 DAS), the HO plants produced more leaves than WT (mean of 18 and 15 leaves, respectively; Figure 1d). Qualitative observation suggested that the greater leaf number of HO plants was associated with a delayed flowering phenotype. Overall, this reduced the difference in aboveground dry biomass at the floral transition with HO plants reaching 60% of WT size (Figure 1b).

Some differences were also observed in leaf morphology. WT leaves were not only larger than HO leaves, but also they were often thicker as indicated by a lower individual‐specific leaf area (SLA) in young (Y) and mature (M) leaves of vegetative stage plants and in young (Y) leaves of floral transition plants (Figure 1e,f). However, this reduction in SLA could also be due to the relatively small size of the HO plants.

Germination, seed size and seed composition were measured to determine whether the observed differences in growth rate could be attributed to these factors. Overall, there was little difference between WT and HO seeds (Table S2). Compared to WT, HO seeds were found to be slightly smaller (5% decrease in seed fresh and dry weight) and to have slightly higher total carbon (1.4% increase). However, there was no difference in seed total fatty acid, TAG, starch, sugar, nitrogen or soluble protein content. Germination index (speed of germination) and hypocotyl length (a measure of seed reserves) were the same for both WT and HO seeds.

### Developmental effects on oil accumulation, carbon and nitrogen partitioning

**Figure 2:**
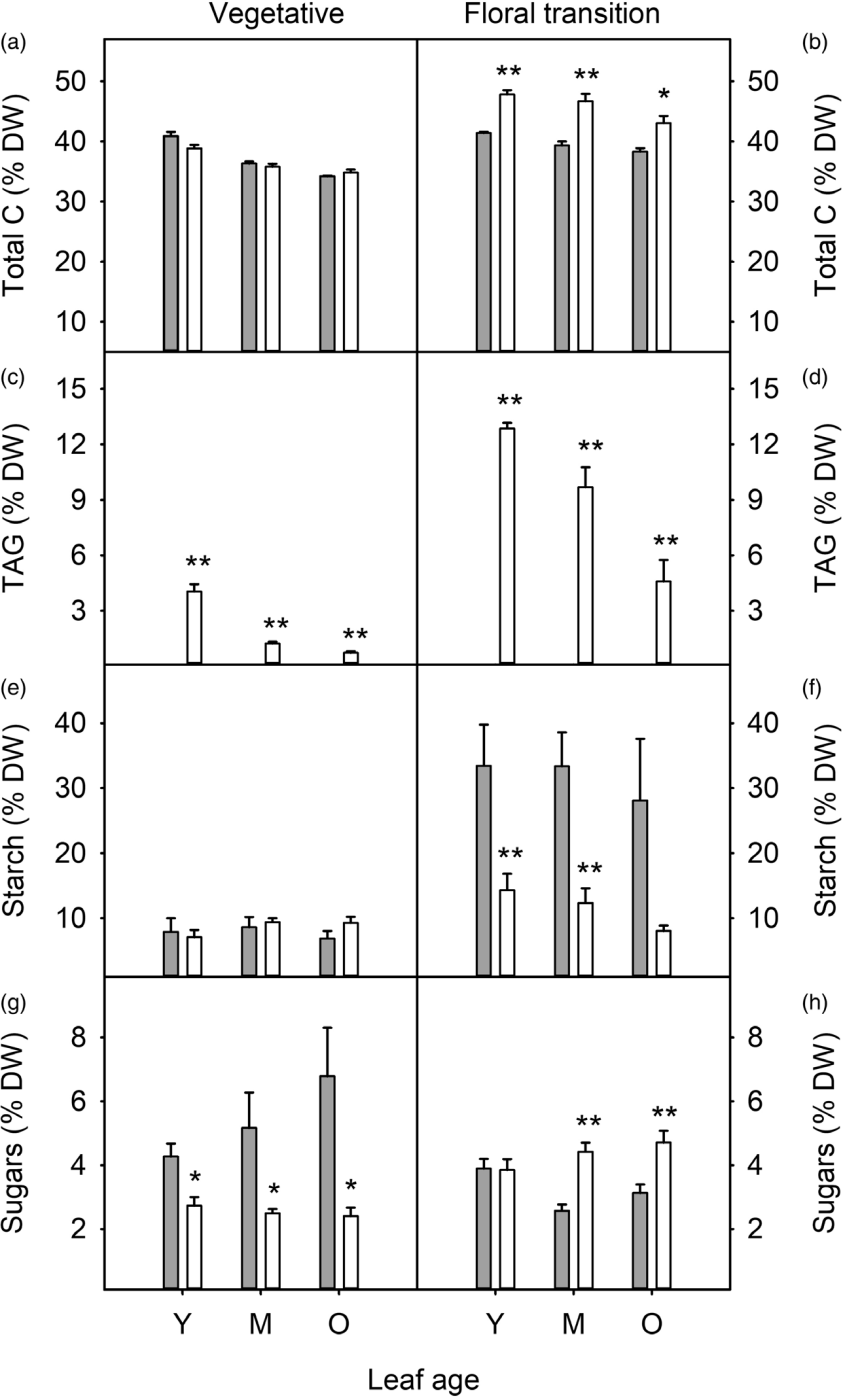
Carbon partitioning during leaf and plant development of WT (grey) and transgenic HO (white) tobacco. Leaf total carbon (a, b), TAG (c, d), starch (e, f) and sugar (g, h) contents are shown for young/expanding (Y), mature/fully expanded (M) and older (O) leaves harvested from plants at the vegetative (a, c, e, g) and floral transition (b, d, f, h) stages of growth. WT TAG is shown but was negligible compared to HO: 0.02–0.04% for vegetative and 0.03–0.11% for floral transition plants. Asterisks represent statistically significant differences (*P < 0.05; **P < 0.01) between the two lines according to a *t* test.

To identify developmental effects on carbon and nitrogen partitioning in WT and HO plants, total carbon, TAG, starch, sugars, total nitrogen, soluble protein and chlorophyll were measured at the end of the day in leaves of three different ages from plants at two different developmental stages. Total carbon content of leaves was 12–19% higher in HO leaves compared with WT at floral transition stage while no difference in total carbon was observed in plants at the vegetative stage (Figure 2a and b). In both vegetative and floral transition HO plants, TAG content was approximately five times higher in younger compared with older leaves (Figure 2c and d). TAG also accumulated during HO plant growth with higher TAG observed in older plants. Leaf starch content in HO lines was reduced to approximately 40% of WT levels at the floral transition stage while no difference in starch content was detected in younger plants (Figure 2e and f). Leaf sugar content was reduced in HO plants at the vegetative stage but was increased in mature and older leaves at the floral transition stage (Figure 2g and h).

**Figure 3:**
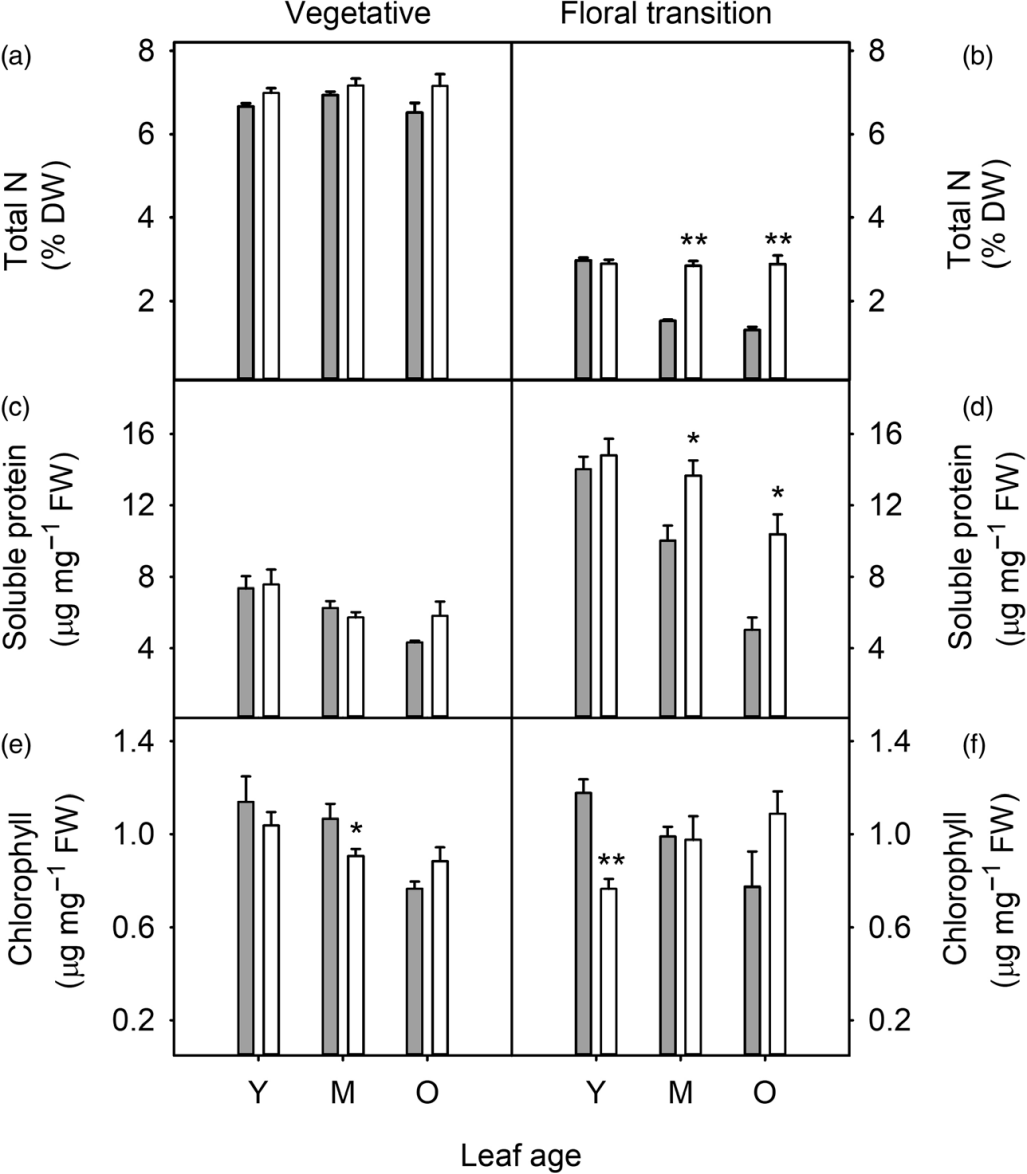
Nitrogen partitioning during leaf and plant development of WT (grey) and transgenic HO (white) tobacco. Leaf total nitrogen (a, b), soluble protein (c, d) and chlorophyll (e, f) contents are shown for young/expanding (Y), mature/fully expanded (M) and older (O) leaves harvested from plants at the vegetative (a, c, e) and floral transition (b, d, f) stages of growth. Asterisks represent statistically significant differences (*P < 0.05; **P < 0.01) between the two lines according to a *t* test.

Nitrogen partitioning was also affected by oil accumulation, particularly in older plants (Figure 3). In HO but not WT plants at the floral transition, total nitrogen and soluble protein were maintained at high levels in mature and older leaves (Figure 3b and d), including the primary photosynthetic carboxylase Rubisco (Figure S2). Chlorophyll concentration was generally similar between WT and HO plants, although chlorophyll content was reduced in young leaves of floral transition HO plants (Figure 3f) and in mature leaves of vegetative plants (Figure 3e).

### Altered carbon partitioning and metabolism during light/dark cycles

**Figure 4:**
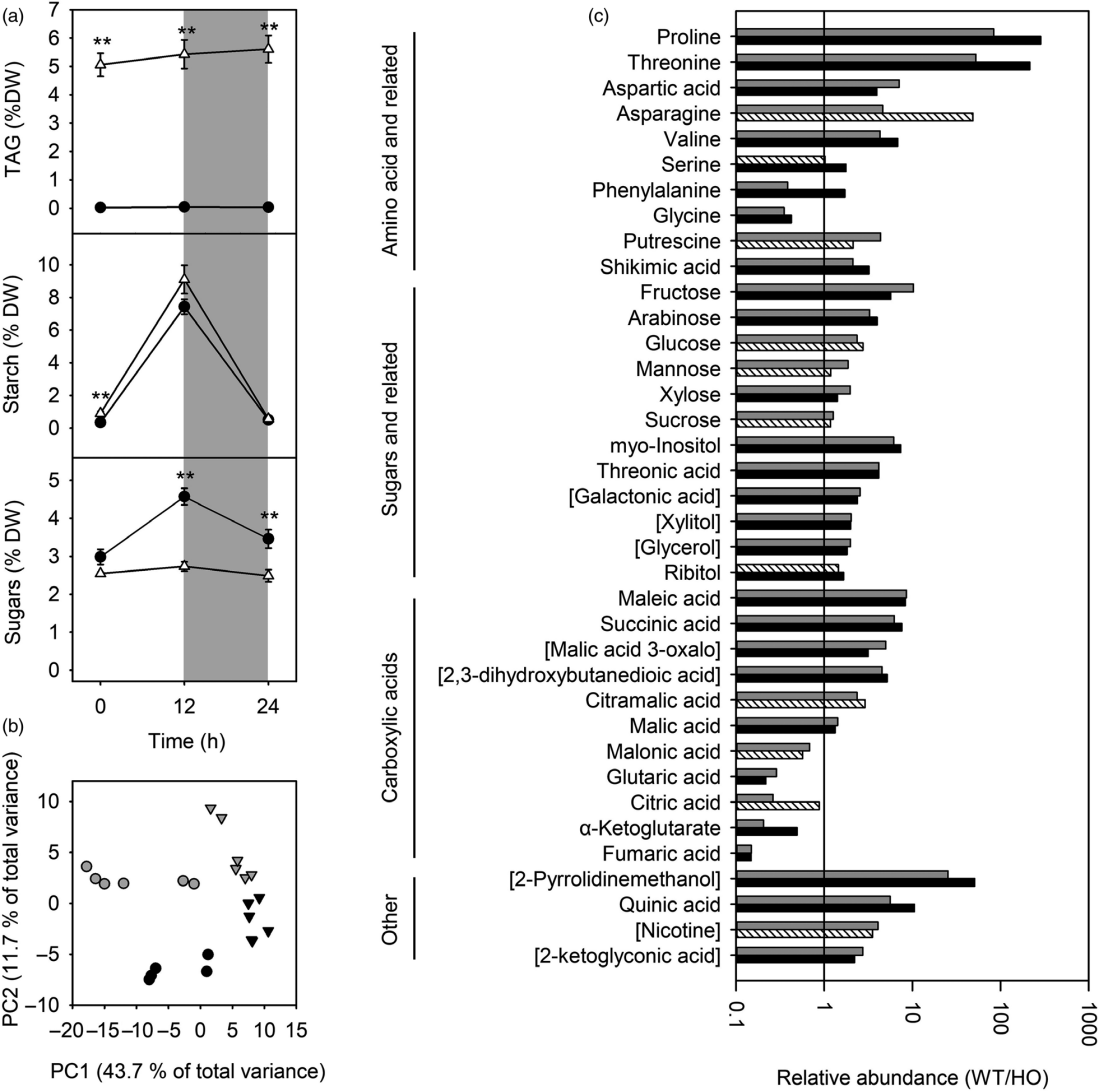
Carbon partitioning and metabolite accumulation in young/expanding leaves from WT and transgenic HO tobacco during a dark/light cycle. Leaf TAG, starch and sugar content in WT (black circles) and HO (white triangles) tobacco (a). Asterisks represent statistically significant differences (**P < 0.01) between the two lines according to a *t* test. Principal components analysis (b) of metabolite abundance in WT (circles) and HO (triangles) plants at the end of the day (grey) and the end of the night (black). Relative abundance of metabolites (c) that are differentially accumulated (*P* < 0.05) at the end of the day (grey) or end of the night (black). Metabolites that are not significantly different between WT and HO are shown using striped bars. Names of metabolites that were only putatively identified are shown in square brackets.

To investigate the relative importance of starch, sugars and TAG as carbon reserves during dark/light cycles, these metabolites were quantified in expanding leaves of vegetative plants over a 24‐h period (Figure 4a). Although TAG comprised a relatively large proportion of leaf carbon/energy reserves, TAG content did not appear to vary during a single dark/light cycle. In contrast, starch accumulated during the day and was almost entirely degraded at night in both WT and HO leaves. Leaf sugars also showed some diurnal variation in WT leaves, with sugars accumulating during the day. HO leaves, on the other hand, did not accumulate sugars during the day leading to reduced sugars compared to WT (Figure 2g).

Analysis by GC‐MS identified distinct polar metabolite profiles separated according to genotype and time of day (Figure 4b). Thirty‐seven metabolites were identified as differentially abundant between WT and HO leaves (Figure 4c). Most of these metabolites were more abundant in WT leaves, including sugars and related compounds as well as amino acids and related compounds. Only two amino acids (glycine and phenylalanine) were more abundant in HO leaves. Carboxylic acids were also differentially accumulated with several being more abundant in HO leaves. Overall, these metabolic profiles indicate changes to the following: the tricarboxylic acid (TCA) cycle; arginine and proline metabolism; alanine, aspartate and glutamate metabolism; phenylalanine metabolism; and glyoxylate and dicarboxylate metabolism. A full list of metabolites and their relative abundance is included in Dataset S1.

### Improved growth and oil yield of supertransformants

**Figure 5:**
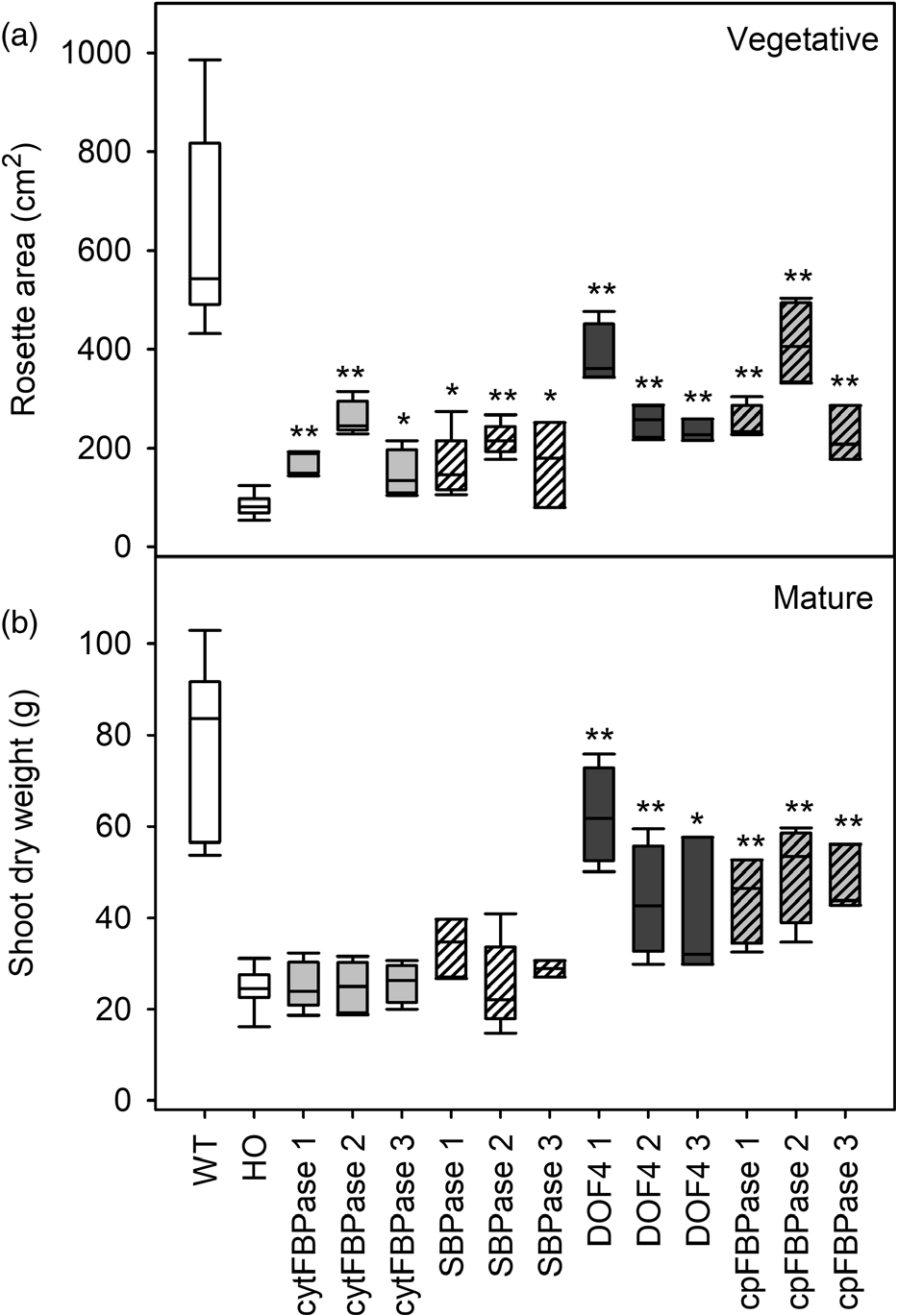
Early growth and final biomass of supertransformants. Vegetative stage rosette area (a) and dry biomass at maturity (b) are shown for WT, HO and three independent events of supertransformant lines. Asterisks indicate significant differences between supertransformant lines and HO control plants as determined by one‐way ANOVA (*P < 0.05; **P < 0.01).

To explore possible means of improving growth and/or oil yields, HO plants were supertransformed with one of the four additional genes to improve carbon supply or partitioning. Overexpression of cytosolic FBPase, SBPase, chloroplast‐targeted FBPase or DOF4 in the HO background improved vegetative growth compared with HO plants, as indicated by greater rosette (leaf) area in seven‐week‐old plants (Figure 5a). Supertransformant rosette area varied from 1.7 to 4.8 times greater than the mean HO rosette area (85 cm^2^), depending on the construct and event. Mean WT area was generally even greater (7.5 times mean HO) than supertransformants, although some individual DOF4 and cpFBPase plants fell within the WT range. The increased rosette area of supertransformants was not associated with changes to leaf TAG or starch content, which remained HO‐like, but was positively correlated with leaf sugar content (Tables S3 and S4).

Final biomass measurements revealed that the vegetative growth advantage was lost later in development for two of the four constructs as the aboveground biomass (leaf plus stem) of SBPase and cytFBPase lines was equivalent to HO plants at seed set (Figure 5b). In contrast, DOF4 and cpFBPase lines had significantly more biomass at seed set than HO plants and their aboveground biomass was positively correlated with transgene copy number (DOF4, *R* = 0.64, *P* = 8.0 × 10^−3^; cpFBPase, *R* = 0.70, *P* = 2.5 × 10^−3^). Mean aboveground biomass was 25 g for HO plants while mean aboveground biomass for DOF4 lines ranged from 40 to 62 g (1.6–2.5 times mean HO) and cpFBPase lines ranged from 45 to 50 g (1.8–2.0 times mean HO). Similar patterns in leaf dry weight were observed across the lines as there was a very strong positive correlation between leaf dry weight and aboveground dry biomass (*R* ^2^ = 0.96, *P* = 2.58 × 10^−56^).

**Figure 6:**
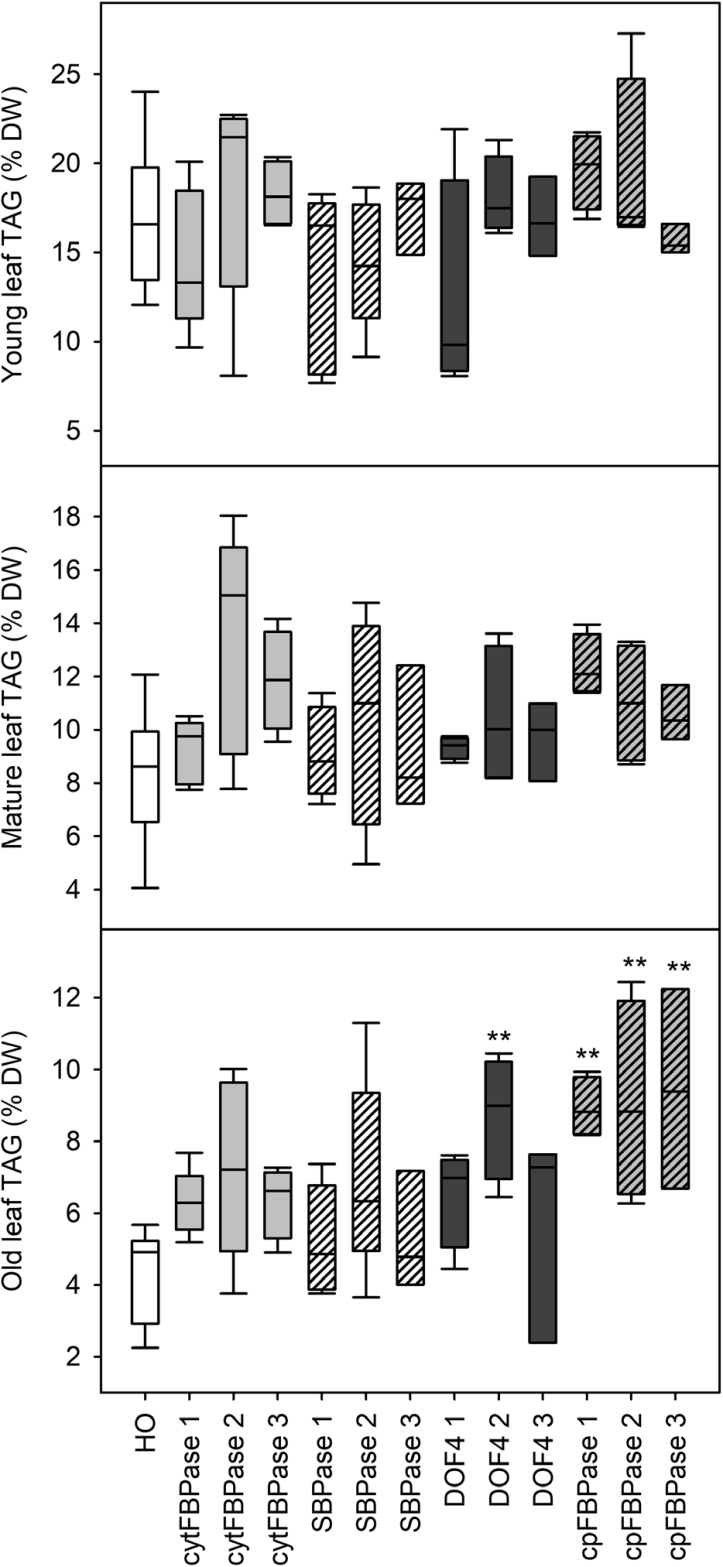
Leaf TAG accumulation in supertransformants. Leaf TAG content is shown for young, mature and old leaves of WT, HO and three independent events of supertransformant lines. Asterisks indicate significant differences between supertransformant lines and HO control plants as determined by one‐way ANOVA (*P < 0.05; **P < 0.01).

**Figure 7:**
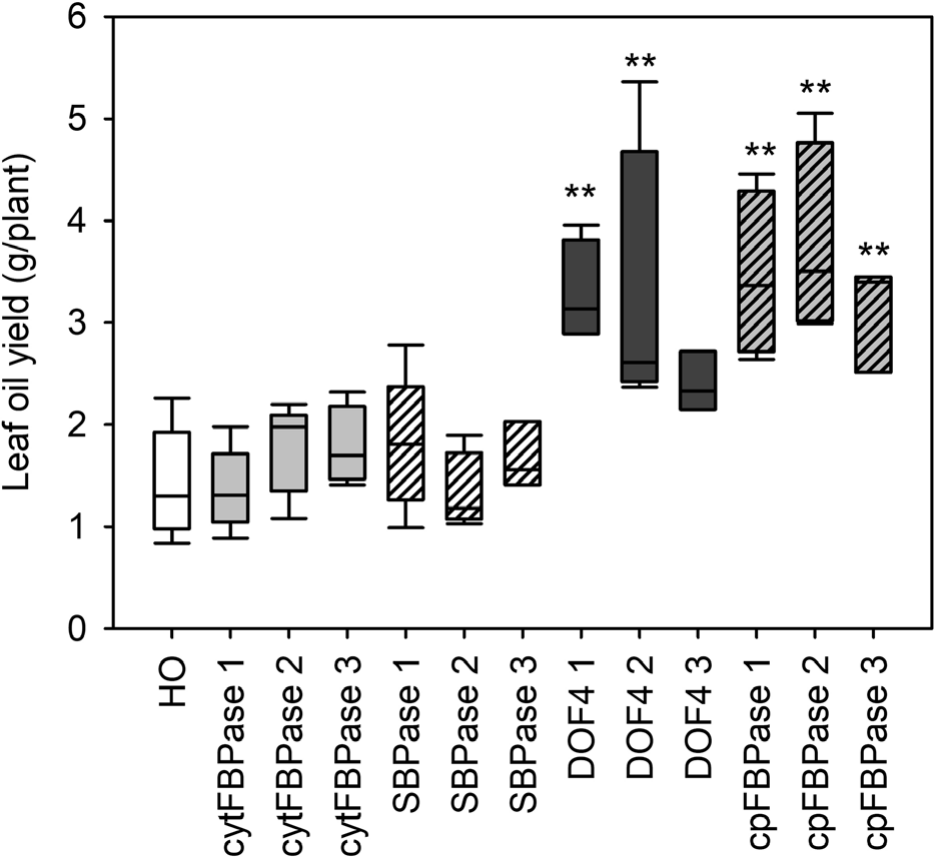
Calculated leaf oil yields of supertransformants. TAG yield per plant was calculated based on measurements of leaf dry biomass and TAG content of leaves of three ages for HO and three independent events of supertransformant lines. Asterisks indicate significant differences between supertransformant lines and HO control plants as determined by one‐way ANOVA (**P < 0.01).

TAG content of leaves of three different ages from plants at the floral transition was also measured and used to predict oil yields. Young leaves appeared to have similar TAG content across all lines while some old leaves appeared to have slightly increased TAG content compared to HO (Figure 6). Overall, large increases in leaf biomass combined with small increases in leaf TAG accumulation led to predicted oil yields in the DOF4 and cpFBPase transgenic lines that were up to three times greater than the mean yield for HO plants (Figure 7). HO leaves were predicted to yield 1–2 g TAG per plant whereas leaves of the supertransformant lines were predicted to yield up to 5.4 g TAG per plant. Similar patterns of aboveground biomass, leaf dry weight and TAG accumulation were observed in the next generation of supertransformed plants (Table S5).

## Discussion

This study has shown that metabolic engineering of plants to accumulate high levels of TAG may include trade‐offs in growth due to competition for carbon and key metabolic precursors. Overall, in HO plants, younger leaves consistently had more TAG than older leaves while leaves of older plants had higher TAG levels than leaves of younger plants, which likely reflects the increased source available for each successive leaf. During both vegetative growth and the transition to flowering, leaf TAG accumulation altered carbon partitioning between lipids, starch and sugars and dramatically reduced plant growth. In the vegetative growth stage, leaf TAG accumulation reached up to 200 times WT levels, seemingly at the expense of sugars and amino acids but not starch. Short day‐grown (carbon‐limited) tobacco also accumulates less sugar, inhibiting amino acid biosynthesis and slowing growth (Matt *et al.*, 1998), which suggests that TAG accumulation might impair the vegetative growth of HO tobacco by reducing the capacity of leaves to accumulate sugars during the day.

These low sugar levels may activate signalling pathways to limit growth in HO plants, despite making a relatively small contribution to the total leaf energy budget. Sugars and sugar phosphates are important signalling molecules as well as substrates for many anabolic and catabolic processes and cellular components. Plants can sense the availability of carbon, including these sugars and sugar phosphates, and alter growth and developmental transitions accordingly. For example, hexokinase 1 (HXK1), target of rapamycin (TOR), Snf1‐related protein kinase 1 (SnRK1) and the phosphorylated sugar trehalose‐6‐phosphate (T6P) are involved in sugar signalling pathways that control growth processes including protein synthesis (Lastdrager *et al.*, 2014). Thus, low sugars may trigger signalling pathways to slow growth even if they do not dramatically affect the amount of carbon available. Indeed, increases in leaf sugar content of supertransformant lines correlated with increased vegetative growth.

Sugar signalling may also delay the development of HO plants as high carbon availability is required for plants to initiate flowering (Bolouri Moghaddam and Van den Ende, 2013; Lastdrager *et al.*, 2014; Wingler, 2018). Notably, the carbohydrate content of leaves from floral transition plants was almost the inverse of vegetative plants; HO leaf starch content was only half of WT levels while sugars were above WT levels in mature and old leaves. These changes may be related to developmental changes associated with the transition to flowering such as starch remobilization, tissue‐specific sugar accumulation and carbohydrate export from the leaves (Bernier *et al.*, 1993; Corbesier *et al.*, 1998; Yu *et al.*, 2000). However, the apparent delay in flowering of HO plants could also be related to delayed leaf senescence and the maintenance of high leaf soluble protein and nitrogen (Weber and Burow, 2018) as well as plant carbon status.

In addition to sugars, other key metabolites and energy stores are affected by TAG accumulation. At the end of the day, HO tobacco has WT‐like starch, low sucrose, glucose, fructose and malic acid but high citrate and succinate, all of which may be converted to energy for growth at night (Pollock, 1986). These changes to carbohydrate and TCA cycle metabolites might reflect different requirements for energy and carbon skeletons in WT and HO leaves in response to major changes in lipid‐related pathways. High citrate and succinate may accumulate via increased fatty acid respiration as well as altered TCA cycle activity and demand for sucrose (Graham, 2008; Troncoso‐Ponce *et al.*, 2013). Thus, despite WT‐like levels of total carbon, vegetative HO plants appear to lack the specific balance of compounds required for WT‐like growth rates. Metabolite labelling and absolute quantification could help to clarify the relative importance and direction of carbon flow in these pathways and identify potential substrate limitations.

It was also hypothesized that TAG might act as an alternative/additional source of carbon to sustain HO plant growth. However, leaf TAG content remained constant over a dark/light cycle, suggesting that TAG is not a net contributor of carbon to night‐time metabolism in these plants. In contrast, slow TAG degradation during the dark period has been shown to improve the growth of starchless *A. thaliana* mutants by supplying fatty acids as an alternative energy source (Fan *et al.*, 2019). Tobacco may thus lack the sensing/metabolic pathways required to fully utilize leaf TAG as an energy source or the WT‐like starch levels in HO leaves may impair this response. In either case, wasteful cycles of TAG synthesis and degradation, as identified by Vanhercke *et al., *(2017), would also compromise TAG as a carbon source.

Promisingly, the limitations to HO plant growth imposed by TAG accumulation were able to be overcome by further engineering of carbon metabolism. Similar to SBPase overexpression in wild‐type tobacco (Lefebvre *et al.*, 2005), SBPase supertransformant lines showed better early growth than HO controls but only minor differences in final biomass. The increased early growth may be linked to increased sugar accumulation while final biomass may be constrained by the availability of other metabolites, other parts of the CBB cycle or regulation of SBPase activity (Raines, 2003; Raines *et al.*, 1999; Stitt and Schulze, 1994). Analogous increases in sugars observed in cytosolic FBPase supertransformant lines may account for increased plant size during early development. Cytosolic FBPase converts triose‐phosphates to sucrose, and FBPase overexpression is linked to increased sucrose and hexose accumulation and source capacity in *A. thaliana* leaves (Cho *et al.*, 2012; Daie, 1993; Otori *et al.*, 2017). As with SBPase, the similarity of the exogenous cytFBPase to native enzymes in tobacco may make it susceptible to post‐translational regulation, for example allosteric regulation by citrate (Van Praag, 1997), resulting in only HO‐like final biomass.

In contrast, supertransformants overexpressing proteins from more distantly related species such as cyanobacterial chloroplast‐targeted FBPase and soya bean DOF4 may not be as affected by gene silencing, post‐translational regulation, redox or other feedback mechanisms (Tamoi *et al.*, 1996; Tamoi *et al.*, 1998), and so are able to translate their early growth advantage into higher final biomass. Similar to SBPase, cpFBPase may boost growth directly by increasing carbon supply and leaf soluble sugar content, through the CBB cycle (Kohler *et al.*, 2017; Miyagawa *et al.*, 2001). The transcription factor DOF4, on the other hand, may have both direct and indirect effects on lipid metabolism, although the specific targets of DOF4 in tobacco leaves remain unknown. Given a general role in up‐regulating lipid metabolism (Wang *et al.*, 2007; Zhang *et al.*, 2014), DOF4 overexpression may increase the availability of carbon for growth by making TAG accumulation more efficient. Constitutive expression of DOF4 may allow supertransformants to synthesize TAG more efficiently during all stages of development and to accumulate much higher biomass while maintaining HO‐like TAG. In contrast, HO plants overexpressing the lipid master regulator, LEC2, under the control of a senescence‐inducible promotor accumulate more oil but less final biomass (Vanhercke *et al.*, 2017), perhaps because early growth is still limited by futile TAG cycling.

This approach of engineering multiple related pathways could be applied to increase the production of other useful products in plants. For example, accumulation of PHA, PHB and cyanophycin polymers can lead to growth retardation in *A. thaliana*, perhaps via competition with plant hormone and sterol pathways (Börnke and Broer, 2010). Lignin reduction also results in a dwarfing phenotype, although it is unclear whether this is due to vasculature defects, altered metabolite levels or feedback from a cell wall integrity signalling pathway (Muro‐Villanueva *et al.*, 2019). In many cases, attempts to engineer plants with increased sugars and simple sugar derivatives have also produced plants with growth and developmental defects (Patrick *et al.*, 2013). In these cases, engineering pathways to produce key substrates for growth—or to reduce the accumulation of intermediates that impair growth—in combination with the metabolic pathway of interest could help to increase yields. Here, it is also worth noting the potential of synthetic biology to increase the speed and throughput of generating/testing various gene combinations and to design entirely novel traits (Medford and McCarthy, 2017).

In conclusion, this study shows that although growth of first‐generation transgenic leaf oil tobacco is carbon‐limited, this may be due to altered carbon partitioning or sugar signalling rather than total carbon reserves. Promisingly, this limitation can be overcome by manipulating carbon fixation or partitioning via the CBB cycle, sucrose and lipid biosynthetic pathways. Individual overexpression of additional genes (cytFBPase, SBPase, cpFBPase and DOF4) in HO tobacco improved early growth while overexpression of cpFBPase or DOF4 also increased final biomass, leading to greater predicted oil yields. Future work will include expressing these and other promising candidate genes in combination to further improve growth and lipid biosynthesis. While agronomic considerations such as timing of harvest and growth conditions will also influence the eventual choice of target genes, this work highlights the potential benefits of engineering multiple pathways to produce novel crops tailored for both high biomass and oil production.

## Methods

### Plant material

#### Wild‐type and high oil plants

For the first part of this study (Figures 1, 2, 3, 4), *Nicotiana tabacum* L. wild‐type (Wisconsin 38, WT) and transgenic high oil (HO) plants overexpressing WRI1 (lipid‐related transcription factor), DGAT1 (lipid biosynthetic enzyme) and oleosin (lipid packaging protein) were used. HO plants were homozygous progeny of a single transformation event that was selected as best and described in a previous study (Vanhercke *et al.*, 2014).

#### Generation and selection of supertransformed HO plants

Genes encoding *A. thaliana* CCB cycle enzyme SBPase (Uniprot ID P46283), *A. thaliana* cytosolic FBPase (Uniprot ID Q9MA79), *Syneccococus elongatus* PCC 7942 FBPase (Uniprot ID Q59943) and *Glycine max* lipid‐related transcription factor DOF4 (Uniprot ID Q0GLE8) were codon‐optimized for expression in *N. tabacum* and synthesized by GeneArt (Thermo Fisher, Waltham, MA, USA). Each of the four genes was individually subcloned into a binary expression vector containing the CaMV‐35S constitutive promoter and hygromycin selectable marker. The transit peptide from the tomato Rubisco small subunit (RBCS1, Uniprot ID P08706) was also codon‐optimized and used to target the cyanobacterial FBPase to the tobacco chloroplast (Miyagawa *et al.*, 2001).

The HO tobacco plants described above were supertransformed with each of the constructs using *Agrobacterium*‐mediated transformation essentially as described by Horsch *et al. *(1985). The *Agrobacterium tumefaciens* strain used was AGL1, and timentin was used to suppress the bacteria after the co‐cultivation. WT and HO plants were also taken through the tissue culture protocol as controls. Progeny of representative WT and HO tissue culture control plants were grown side‐by‐side with each generation of supertransformant plants.

Progeny of three independent transgenic events per construct were analysed, and plants in each generation were screened for copy number by digital droplet PCR using probes for the selectable marker gene as described in Vanhercke *et al. *(2017). Due to space limitations, it was not possible to identify three independent stable lines for all four constructs. Stable/homozygous lines that were analysed included: cytFBPase 1, 2 and 3; SBPase 1 and 2; DOF4 1 and 2; and cpFBPase 3. Plants from the other lines (SBPase 3, DOF4 3 and cpFBPase 1 and 2) were confirmed as positive for transgene copy number prior to analysis. The main study presents data from SBPase and cytFBPase plants of the T2 generation and cpFBPase and DOF4 plants of the T1 generation. The Supplementary Material includes data confirming the phenotypes in subsequent generations (T3 SBPase and cytFBPase, T2 cpFBPase and DOF4). Expression of transgenes was confirmed by RT‐PCR (Figure S1) and primer sequences are included in Table S1.

### Growth conditions

For experiments comparing WT and HO plants, plants were grown together in Conviron PGC20FLEX growth cabinets (Controlled Environments, Winnipeg, Canada). Plants were grown in 12 h:12 h light/dark cycles (25 °C day : 20 °C night) with 300 µmol photons m^−2^ s^−1^ illumination from fluorescent lights. Plants were grown in soil supplemented with slow release fertilizer (Osmocote all purpose, Scotts Australia, Bella Vista, Australia) and were watered once per day.

Supertransformant lines, WT and HO plants were grown together in the glasshouse at 24°C with natural light. Plants were grown in soil supplemented with slow release fertilizer (as above) and were watered once or twice per day, depending on plant size.

### Relative growth rate, leaf area and aboveground biomass

Growth rate was determined by measuring the aboveground dry weight of a minimum of five (WT or HO) plants harvested during the vegetative growth phase at 21, 25, 28, 32, 34 and 39 days after sowing (DAS). Leaf area was measured using a CI‐203 leaf area metre with CI‐203CA conveyor attachment (CID BioScience, Camas, WA, USA). Plant size at the vegetative stage was determined from photographs by measuring rosette area using ImageJ (Schneider *et al.*, 2012). Leaves and stems were harvested and dried at 70°C for at least 72 h to determine aboveground biomass.

### Carbon and nitrogen partitioning

For determining the effects of leaf and plant age on carbon and nitrogen partitioning, leaves of different ages were harvested from WT and HO plants at the end of the day at the vegetative or floral transition stage (six and ten weeks after sowing, respectively). From top to bottom of the plant, the leaves were defined as: young or still expanding (Y); mature or the first fully expanded leaf (M); and older leaves several nodes below mature leaves (O). For carbon partitioning during light/dark cycles, expanding leaves were harvested six weeks after sowing at the end of the first night, the end of the following day and the end of the second night. For TAG, starch, sugar, total carbon, total nitrogen, chlorophyll and soluble protein analyses, leaf discs were snap frozen in liquid nitrogen, freeze‐dried and homogenized using a tissue lyser. For analysis of other metabolites, expanding leaves were harvested six weeks after sowing at the end of the day and at the end of the following night. Metabolite samples were snap frozen in liquid nitrogen and homogenized while frozen.

Total carbon and nitrogen in freeze‐dried leaf tissue were determined by mass spectrometry (MS) as described in Vanhercke *et al. *(2017). Chlorophyll was quantified spectrophotometrically according to Cross *et al. *(2006). Soluble protein was quantified using Bradford reagent (Bio‐Rad, Hercules, CA, USA) according to manufacturer's instructions.

For quantification of TAG content, a chloroform : methanol : 0.1 M KCl (2 : 1 : 1 [v/v/v]) extraction was added to freeze‐dried tissue (30 mg) as described in Vanhercke *et al. *(2014). Lipids were extracted into the lower phase while sugars were extracted into the upper phase and starch remained in the solid interphase. All three fractions were kept for subsequent analysis. TAG was quantified by thin layer chromatography separation, fatty acid methylation and gas chromatography (GC) as previously described (Vanhercke *et al.*, 2014). For total soluble sugars, aliquots of the upper phase were boiled in anthrone reagent (0.2% [w/v] anthrone in 70% [v/v] concentrated H_2_SO_4_) for 10 min and the absorbance was measured at 630 nm (Yemm and Willis, 1954). For starch determination, the interphase pellet was resuspended in 350 µl 0.2 M NaOH, boiled for 30 min and neutralized using 3.5 µl glacial acetic acid. The starch content of duplicate 100 µl aliquots was determined relative to a control/blank aliquot using a Megazyme Total Starch Kit (Megazyme International, Wicklow, Ireland).

Metabolites were extracted from frozen, homogenized tissue (approximately 100 mg) by incubation at 60 °C for 15 min in five volumes of methanol : H_2_O (20 : 3 [v/v]) containing sorbitol (8.7 µg ml^−1^) as an internal standard. Cell debris was pelleted by centrifugation at > 10 000 ***g*** for 10 min at 20 °C. The supernatant was derivatized and analysed by GC‐MS as originally described by Roessner *et al. *(2000) with modifications described in Mitchell *et al. *(2017). MetabolomeExpress (Carroll *et al.*, 2010) was used to process raw GC‐MS data and conduct statistical analysis including principal components analysis.

### Experimental design and data analysis

WT and HO plants were grown in an alternating layout in the growth cabinet and spaced to minimize shading of upper leaves. Supertransformed plants were arranged in a randomized block design in the glasshouse. WT and HO untransformed tissue culture control plants of the same ‘generation’ were grown alongside supertransformed plants.

All analyses were performed on at least three replicate plants of each genotype. Statistical analyses were performed using SigmaPlot 14.0 (*t* test, ANOVA; Systat Software) or Microsoft Excel (regression).

## Conflict of interests

The authors declare that they have no competing interests.

## Author contributions

MM conceived of the study, designed the experiments, performed experiments and wrote the manuscript. MM, JP, SO, IV and JZ performed experiments and analysed data. TV provided WT and HO tobacco seed and contributed to the conception, design and analysis of the study. JPR contributed to the conception, design and analysis of the study. All authors read and approved the manuscript.

## Supporting information


**Figure S1 **Transgene expression levels in supertransformant lines as determined by RT‐PCR.
**Figure S2 **SDS‐PAGE analysis of protein from young, mature and old leaves of wild‐type and transgenic high oil tobacco plants.
**Table S1 **Primers used to determine transgene expression in supertransformant lines.
**Table S2 **Size, composition and germination of wild‐type and transgenic high oil tobacco seed.
**Table S3 **TAG, starch and sugar content of young leaves of vegetative stage high oil and supertransformant lines.
**Table S4 **Correlations between early plant growth, leaf sugar content and transgene copy number in supertransformant lines.
**Table S5 **Final biomass, leaf TAG content and predicted oil yields of high oil and supertransformant lines.

**Dataset S1 **Metabolites identified in young leaves from vegetative wild‐type and high oil plants harvested at the end of the day and the end of the night.
