# Supplementary material for: Increasing growth and yield by altering carbon metabolism in a transgenic leaf oil crop
Source: Plant Biotechnol J. 2020 Mar 18;18(10):2042–52. doi: 10.1111/pbi.13363 (PMC7539989; doi:10.1111/pbi.13363)
Supplement: Supplementary file 1 — Figure S1 Transgene expression levels in supertransformant lines as determined by RT‐PCR. Figure S2 SDS‐PAGE analysis of protein from young, mature and old leaves of wild‐type and transgenic high oil tobacco plants. Table S1 Primers used to determine transgene expression in supertransformant lines. Table S2 Size, composition and germination of wild‐type and transgenic high oil tobacco seed. Table S3 TAG, starch and sugar content of young leaves of vegetative stage high oil and supertransformant lines. Table S4 Correlations between early plant growth, leaf sugar content and transgene copy number in supertransformant lines. Table S5 Final biomass, leaf TAG content and predicted oil yields of high oil and supertransformant lines. [file PBI-18-2042-s002.docx]

**Increasing growth and yield by altering carbon metabolism in a transgenic leaf oil crop**

Mitchell et al.

# Supplementary material

#### Supplementary Dataset S1

Metabolites identified in young leaves from vegetative WT and HO plants harvested at the end of the day and the end of the night. Attached as separate Excel file.

#### Supplementary Table S1.

Primers used to determine transgene expression in supertransformant lines. Primers were designed using NCBI Primer-BLAST (Ye et al., 2012) to be specific to the transgene.

| **Primer name** | **Sequence (5’ to 3’)** |
| --- | --- |
| cytFBPase F | AAGCTTAGGGTGTTGCACGA |
| cytFBPase R | GGAAGATTGGAGCCCTCTCG |
| SBPase F | GAGGCTCTCCAGTACTCCCA |
| SBPase R | GCTTATCACCTGGCCACACT |
| cpFBPase F | CTCGATGGCTCCGCTAATGT |
| cpFBPase R | CAGCAGCGATCTGCCTATCA |
| DOF4 F | TGAGGCTGTTTCTGCTCCAG |
| DOF4 R | GGATCCGAGTGGCTCGTTAG |

#### Supplementary Table S2.

Size, composition and germination of WT and transgenic HO tobacco seed. Statistical significance was determined using a t-test. For germination index, aliquots of seeds were surface-sterilised and plated onto agarose plates. Seeds were stratified in the dark at 4 °C for 3 d before being incubated in continuous light at 22 °C. Radicle emergence was monitored and used to calculate germination index. For hypocotyl length, stratified seeds were exposed to light for 1 h before the plates were incubated vertically in the dark at 22 °C. Plates were imaged after seven days and hypocotyl length was measured using ImageJ (Schneider et al., 2012).

|  | | **WT** | **HO** | **p-value**  **(n)** |
| --- | --- | --- | --- | --- |
|  |  | Mean ± SEM | |  |
| **Seed size** | Fresh weight (µg) | 77.6 ± 0.5 | 74.5 ± 0.5 | <0.01 (20) |
|  | Dry weight (µg) | 71.7 ± 0.7 | 68.1 ± 0.9 | <0.01 (20) |
|  | Water content | 7.62 ± 0.43 | 7.35 ± 0.38 | ns (4) |
| **Seed composition** | Total C | 56.7 ± 0.14 | 57.5 ± 0.09 | <0.01 (5) |
| (% dry weight) | Fatty acids | 30.5 ± 1.3 | 31.4 ± 0.6 | ns (5) |
|  | TAG | 26.6 ± 1.4 | 28.4 ± 0.9 | ns (5) |
|  | Starch | 0.081 ± 0.005 | 0.085 ± 0.004 | ns (5) |
|  | Soluble sugars | 2.69 ± 0.09 | 2.60 ± 0.14 | ns (5) |
|  | Total N | 3.54 ± 0.024 | 3.60 ± 0.018 | ns (4) |
|  | Soluble protein | 2.67 ± 0.09 | 2.60 ± 0.14 | ns (5) |
| **Germination** | Germination index | 1.32 ± 0.06 | 1.26 ± 0.05 | ns (150) |
|  | Hypocotyl length (mm) | 77.7 ± 2.4 | 80.3 ± 2.2 | ns (115) |

#### Supplementary Table S3.

TAG, starch and sugar content of young leaves of vegetative stage high oil (HO) and supertransformant lines. Four plants from three independent events per construct were analysed.

|  | **TAG (% DW)** | | **Starch (% DW)** | | **Sugars (% DW)** | |
| --- | --- | --- | --- | --- | --- | --- |
|  | **mean** | **SEM** | **mean** | **SEM** | **mean** | **SEM** |
| HO (equivalent cytFBPase, SBPase T2) | 5.33 | 0.74 | 4.91 | 1.37 | 4.04 | 0.35 |
| cytFBPase 1 | 3.59 | 1.48 | 2.79 | 0.96 | 4.27 | 0.39 |
| cytFBPase 2 | 2.87 | 0.60 | 5.45 | 1.53 | 4.65 | 0.16 |
| cytFBPase 3 | 5.37 | 0.44 | 3.84 | 1.00 | 3.99 | 0.17 |
| SBPase 1 | 4.45 | 0.13 | 5.27 | 0.93 | 3.09 | 0.22 |
| SBPase 2 | 5.88 | 0.14 | 3.46 | 0.47 | 4.15 | 0.12 |
| SBPase 3 | 4.99 | 0.77 | 3.24 | 0.52 | 3.84 | 0.58 |
| HO (equivalent DOF4, cpFBPase T1) | 6.32 | 0.90 | 10.10 | 0.76 | 2.98 | 0.43 |
| DOF4 1 | 7.51 | 0.56 | 12.74 | 1.36 | 3.47 | 0.38 |
| DOF4 2 | 6.31 | 0.83 | 10.20 | 0.36 | 2.73 | 0.32 |
| DOF4 3 | 6.24 | 0.53 | 11.32 | 0.69 | 3.34 | 0.40 |
| cpFBPase 1 | 9.06 | 1.94 | 11.60 | 0.68 | 3.35 | 0.22 |
| cpFBPase 2 | 7.89 | 1.15 | 9.94 | 1.20 | 3.25 | 0.05 |
| cpFBPase 3 | 10.22 | 1.47 | 9.04 | 0.60 | 2.87 | 0.45 |

#### Supplementary Table S4

Correlations between early plant growth (rosette area at the vegetative stage), leaf sugar content (% DW) and transgene copy number in supertransformant lines.

|  | **Rosette area vs leaf sugar** | | **Rosette area vs copy number** | |
| --- | --- | --- | --- | --- |
|  | **R^2^** | **p-value** | **R^2^** | **p-value** |
| cytFBPase (T2) | 0.73 | 2.4 x 10^-4^ | 0.76 | 9.3 x 10^-5^ |
| SBPase (T2) | 0.70 | 5.9 x 10^-4^ | 0.64 | 2.2 x 10^-3^ |
| cpFBPase (T1) | 0.51 | 0.022 | 0.76 | 7.0 x 10^-4^ |
| DOF4 (T1) | 0.46 | 0.043 | 0.12 | 0.19 |

#### Supplementary Table S5.

Final biomass, leaf TAG content and predicted oil yields of high oil (HO) and supertransformant lines (n = 5). Asterisks indicate significant difference in shoot DW and oil yield between supertransformant lines and HO as determined by one-way ANOVA (*p < 0.05; **p < 0.01).

|  | **Shoot DW** | | **Young leaf TAG (% DW)** | | **Mature leaf TAG (% DW)** | | **Old leaf TAG (% DW)** | | **Leaf oil yield (g/plant)** | |
| --- | --- | --- | --- | --- | --- | --- | --- | --- | --- | --- |
|  | **mean** | **SEM** | **mean** | **SEM** | **mean** | **SEM** | **mean** | **SEM** | **mean** | **SEM** |
| HO (equivalent cytFBPase, SBPase T3) | 30.4 | 3.6 | 17.8 | 0.3 | 13.1 | 0.5 | 5.5 | 0.7 | 2.4 | 0.3 |
| cytFBPase 4 | 43.9** | 3.8 | 16.8 | 1.0 | 14.2 | 1.4 | 10.0 | 1.0 | 3.6 | 0.5 |
| cytFBPase 5 | 39.8 | 1.8 | 16.2 | 0.4 | 15.4 | 0.7 | 11.5 | 0.8 | 3.5 | 0.2 |
| cytFBPase 6 | 30.3 | 1.6 | 15.8 | 2.3 | 10.3 | 2.1 | 4.5 | 1.4 | 1.9 | 0.5 |
| SBPase 4 | 39.6** | 2.2 | 16.6 | 1.0 | 14.7 | 0.9 | 9.4 | 1.0 | 3.4 | 0.4 |
| SBPase 5 | 45.3** | 3.9 | 13.0 | 0.9 | 13.1 | 0.7 | 7.3 | 0.7 | 3.0 | 0.3 |
| HO (equivalent DOF4, cpFBPase T2) | 28.7 | 2.4 | 16.1 | 1.1 | 11.0 | 0.5 | 4.5 | 0.4 | 1.9 | 0.3 |
| DOF4 4 | 54.7** | 3.5 | 14.5 | 1.0 | 9.8 | 0.6 | 6.9 | 0.4 | 3.0* | 0.3 |
| DOF4 5 | 57.8** | 3.8 | 15.5 | 0.3 | 12.0 | 0.9 | 8.6 | 0.8 | 3.9** | 0.5 |
| DOF4 6 | 57.5** | 3.2 | 15.2 | 0.5 | 12.7 | 1.2 | 10.2 | 1.4 | 4.2** | 0.3 |
| cpFBPase 4 | 50.6** | 2.9 | 15.8 | 0.7 | 13.3 | 0.8 | 9.0 | 0.8 | 3.8** | 0.2 |
| cpFBPase 5 | 50.0** | 2.1 | 16.6 | 0.3 | 13.6 | 0.9 | 8.8 | 0.8 | 3.9** | 0.2 |
| cpFBPase 6 | 51.6** | 2.6 | 15.0 | 0.7 | 10.8 | 0.8 | 7.8 | 0.8 | 3.2** | 0.3 |

#### Supplementary Figure S1.

Transgene expression levels in supertransformant lines as determined by RT-PCR. RNA was extracted using a Maxwell RSC Instrument and Maxwell RSC Plant RNA Kit (Promega, Madison WI, USA). cDNA was synthesised using the SensiFAST cDNA Synthesis Kit (Bioline, UK) and PCRs were performed using GoTaq Flexi DNA polymerase (Promega). Results are shown from a screen of three independent transformation events per construct and 4-5 plants per line. Transgene copy number (using probes for the selectable marker) is shown above each gel. SBPase and cytFBPase plants were from the T3 generation while DOF4 and cpFBPase were from the T2 generation. Control primers amplified a gene encoding the L25 ribosomal protein gene (Schmidt and Delaney, 2010). Wildtype (W) and high oil (H) controls are shown in the four right-hand lanes. Note that cpFBPase plants positive for copy number but with low/no expression are from the same parent, suggestive of gene silencing.


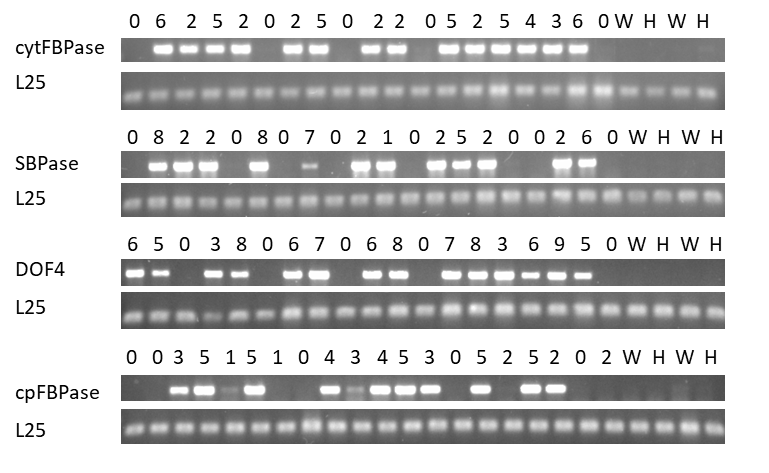


#### Supplementary Figure S2.

SDS-PAGE analysis of protein from young (Y), mature (M) and old (O) leaves of wild-type (WT) and transgenic high oil (HO) tobacco plants. Proteins were extracted in buffer (100 mM Tris pH 8.0, 10 mM MgCl_2_), quantified using Bradford reagent and gel loading was normalised by protein amount (20 µg). Molecular weight marker (MWM) is shown in the far left lane. Rubisco large subunit (53 kDa) is indicated by an arrow.


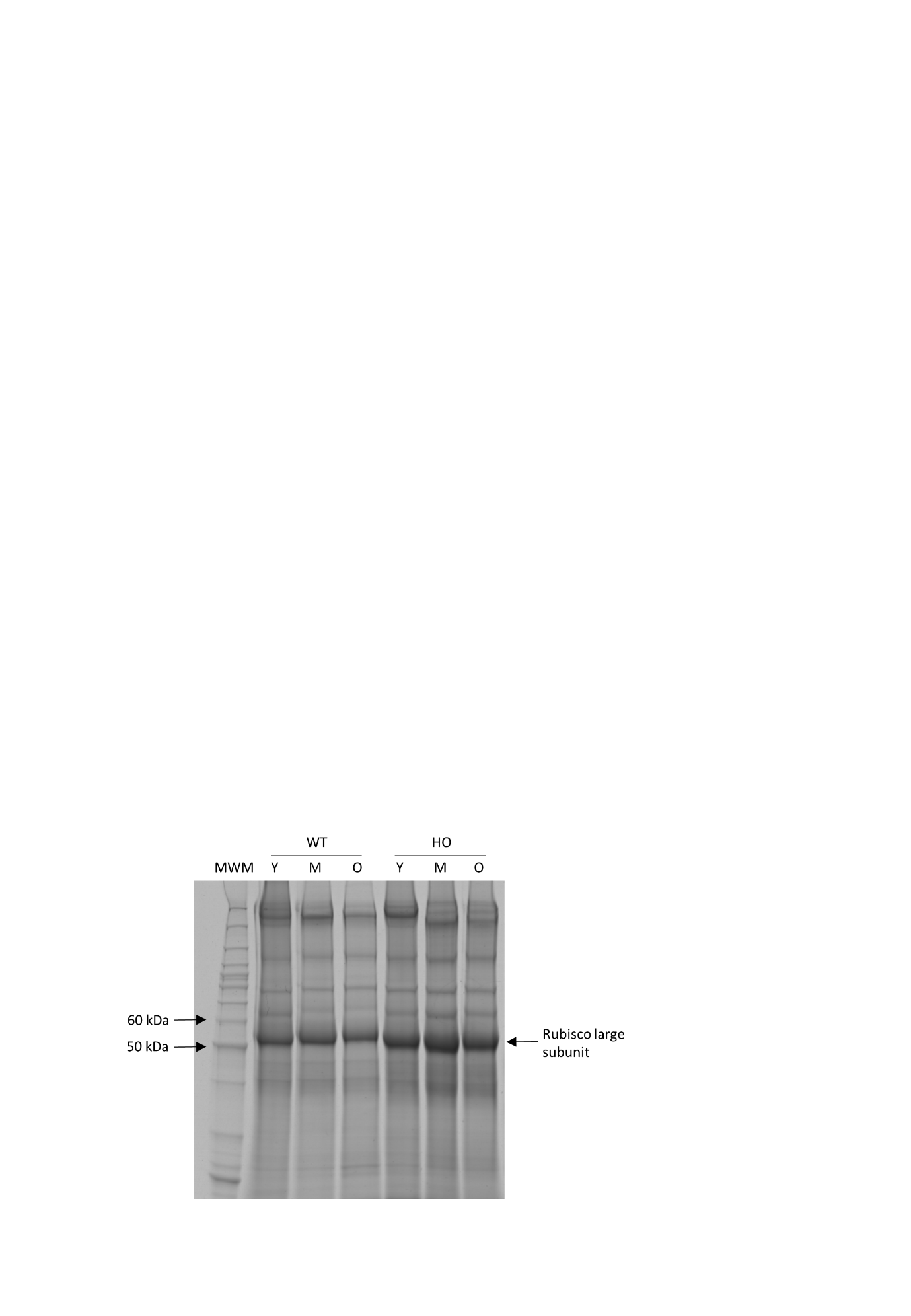


**Supplementary material references**

Schmidt, G.W. and Delaney, S.K. (2010) Stable internal reference genes for normalization of real-time RT-PCR in tobacco (*Nicotiana tabacum*) during development and abiotic stress. *Molecular Genetics and Genomics* 283, 233-241.

Ye, J., G. Coulouris, I. Zaretskaya, I. Cutcutache, S. Rozen and T. L. Madden (2012). Primer-BLAST: A tool to design target-specific primers for polymerase chain reaction. *BMC Bioinformatics* 13.
